# Supplementary material for: Cancer-associated mutations in DICER1 RNase IIIa and IIIb domains exert similar effects on miRNA biogenesis
Source: Nat Commun. 2019 Aug 15;10:3682. doi: 10.1038/s41467-019-11610-1 (PMC6695490; doi:10.1038/s41467-019-11610-1)
Supplement: Supplementary file 1 — Supplementary Information [file 41467_2019_11610_MOESM1_ESM.pdf]

Supplementary Information for Vedanayagam et al,

Cancer-associated mutations in DICER1 RNase IIIa and IIIb domains exert similar effects on miRNA biogenesis

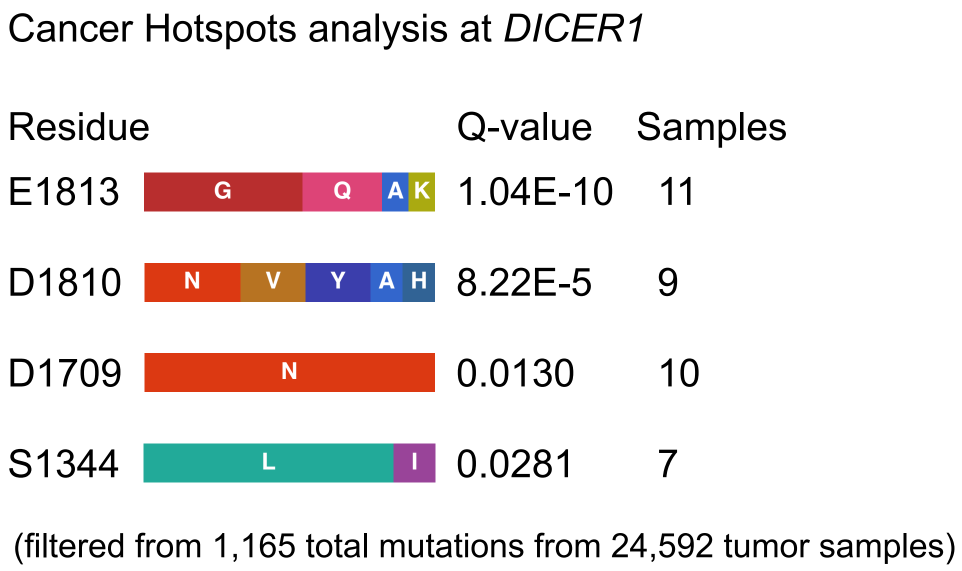

Supplementary Figure 1. Both RNase IIIa and RNase IIIb domains of DICER1 harbor statistically significant hotspot mutations in cancer. Allele distributions and Q-values of three RNase IIIb hotspot residues (E1813, E1810 and D1709) and an uncharacterized RNase IIIa hotspot residue (S1344), from https://www.cancerhotspots.org.

Vedanayagam et al
Supplementary Figure 1

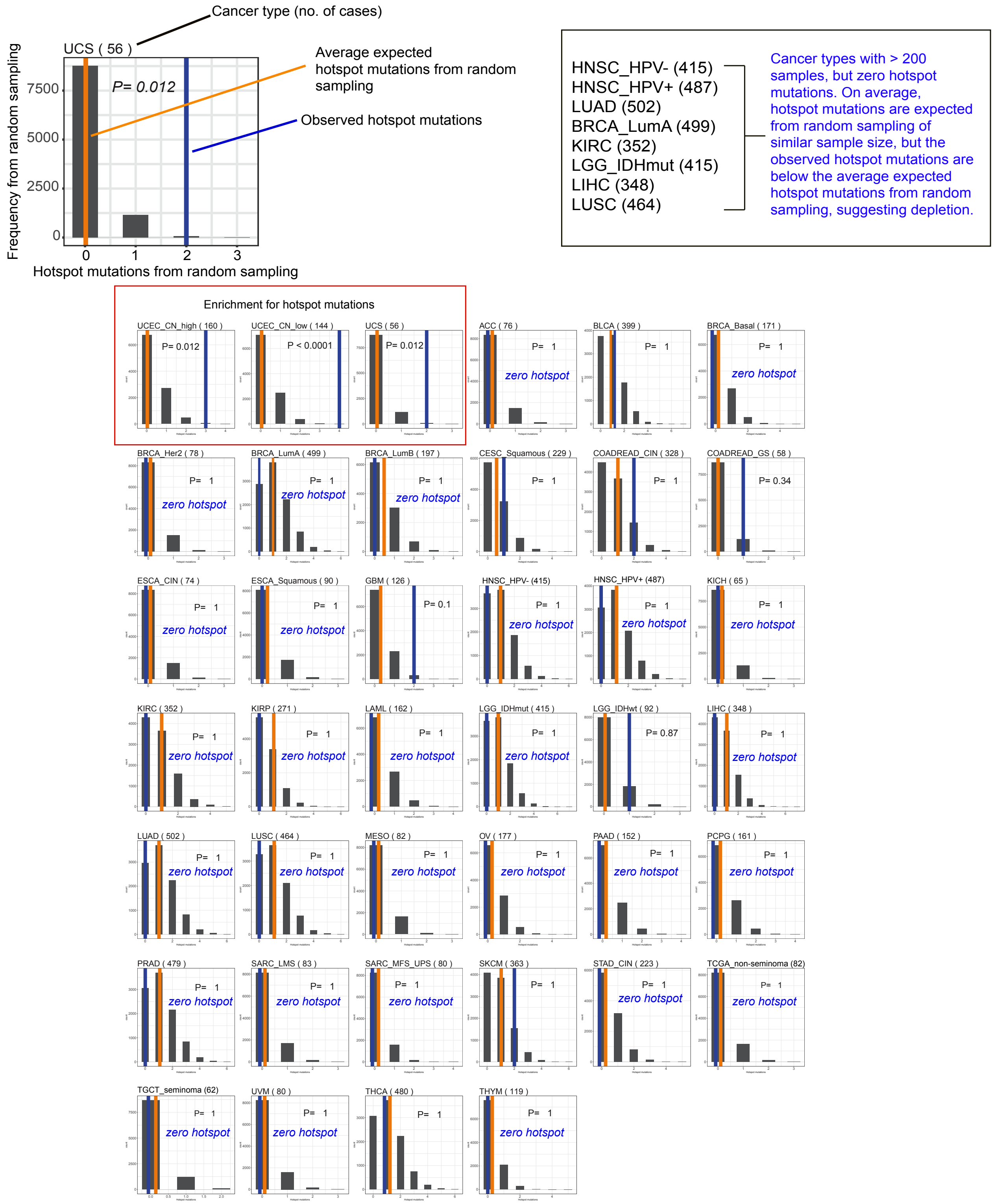

Supplementary Figure 2: Bootstrap resampling analysis for enrichment of hotspot mutations in TCGA PanCan data. Three Uterine cancer types, Uterine Corpus Endometrial Carcinoma (CN high and CN low), and uterine carcinosarcoma are enriched for hotspot mutations (red box). For every cancer type, frequency of hotspot mutations observed from random sampling is shown as a histogram (grey bars). The average expected hotspot mutations from 10000 bootstrap replicates is shown (orange line), along with the actual observed hotspot mutations for every TCGA cancer type (blue line). TCGA cancer types with zero hotspot mutations are highlighted and these make up 70% of TCGA cancer types with > 50 samples. Cancer types with sample size > 200 shown in the box on the right corner illustrates dearth of hotspot mutations in cancers that have similar or higher samples compared to hotspot enriched uterine cancers, indicating depletion of hotspot mutations for most TCGA cancer types. Note that for all cases with zero hotspot mutations, we were not able to estimate statistical significance for depletion due to highly left skewed distribution from resampling, as a result of overall low abundance of hotspot mutations in TCGA PanCan data (31 out of 9919 samples). However, for all these cases, the observed hotspot mutations (blue line) are well below the average hotspot mutations expected from 10000 bootstrap replicates.

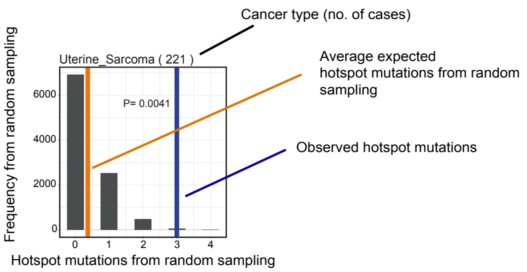

(A)

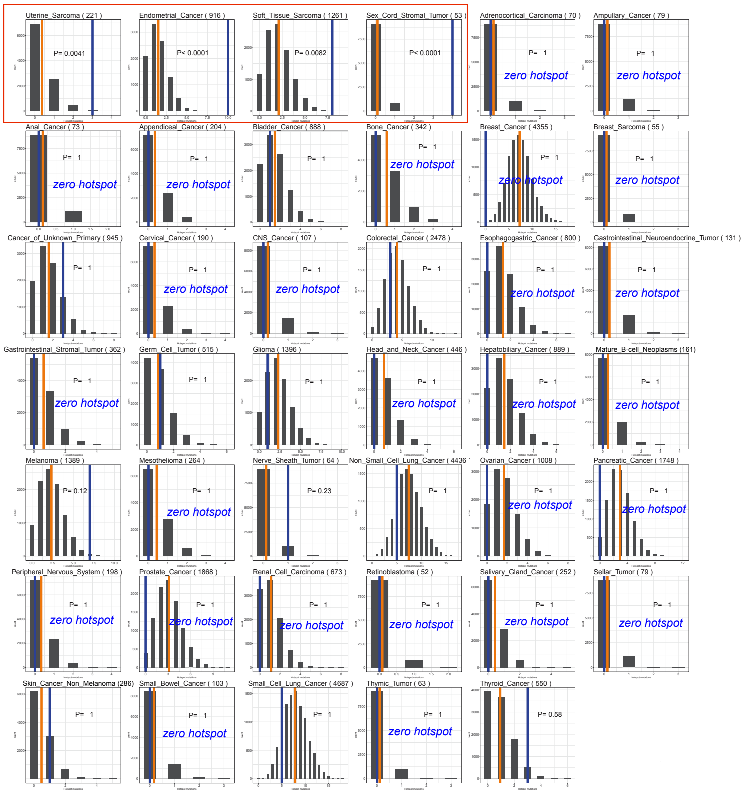

(B)

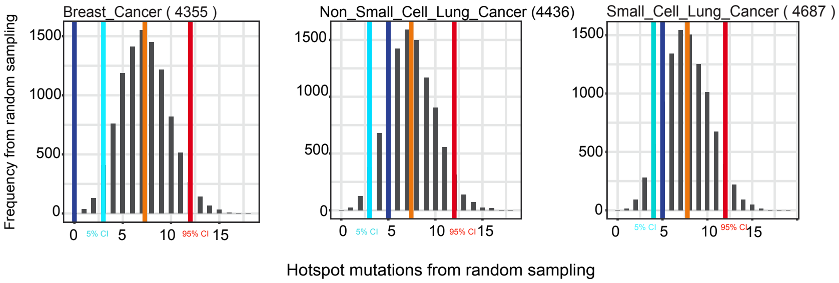

(C)

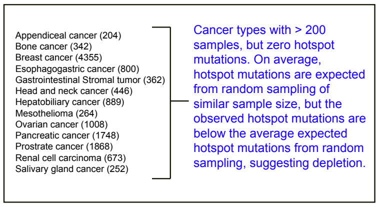

Supplementary Figure 3: Resampling analysis for enrichment of hotspot mutations in MSK-IMPACT cancer types. (A) For every cancer type, frequency of hotspot mutations observed from random sampling is shown as a histogram (grey bars). The orange line shows the average hotspot mutations observed from 10000 bootstrap replicates, and the blue line shows observed hotspot mutations in MSK-IMPACT data. Cancer types with zero observed hotspot mutations are highlighted, for which significance for depletion cannot be computed. 2 Uterine cancer types; sarcoma and sex cord stromal tumor are enriched for hotspot mutations (red box). (B) For three cancer types, breast cancer, Non-small cell lung cancer, and Small cell lung cancer, bootstrap resampling generateds a normal distribution where the percentile confidence intervals for the variance statistic can be computed (5% and 95% confidence intervals). For breast cancer, the observed *DICER1* hotspot mutation is below 5% CI, indicating statistically significant depletion for this cancer type ($P < 0.05$). (C) 13 MSK-IMPACT cancer types with sample sizes > 200, larger than typical uterine cancer cohorts with clear hotspot enrichment,but lacking *DICER1* hotspot mutations, indicating overall dearth of hotspot mutations across the vast majority of cancer types with considerably large sample sizes.

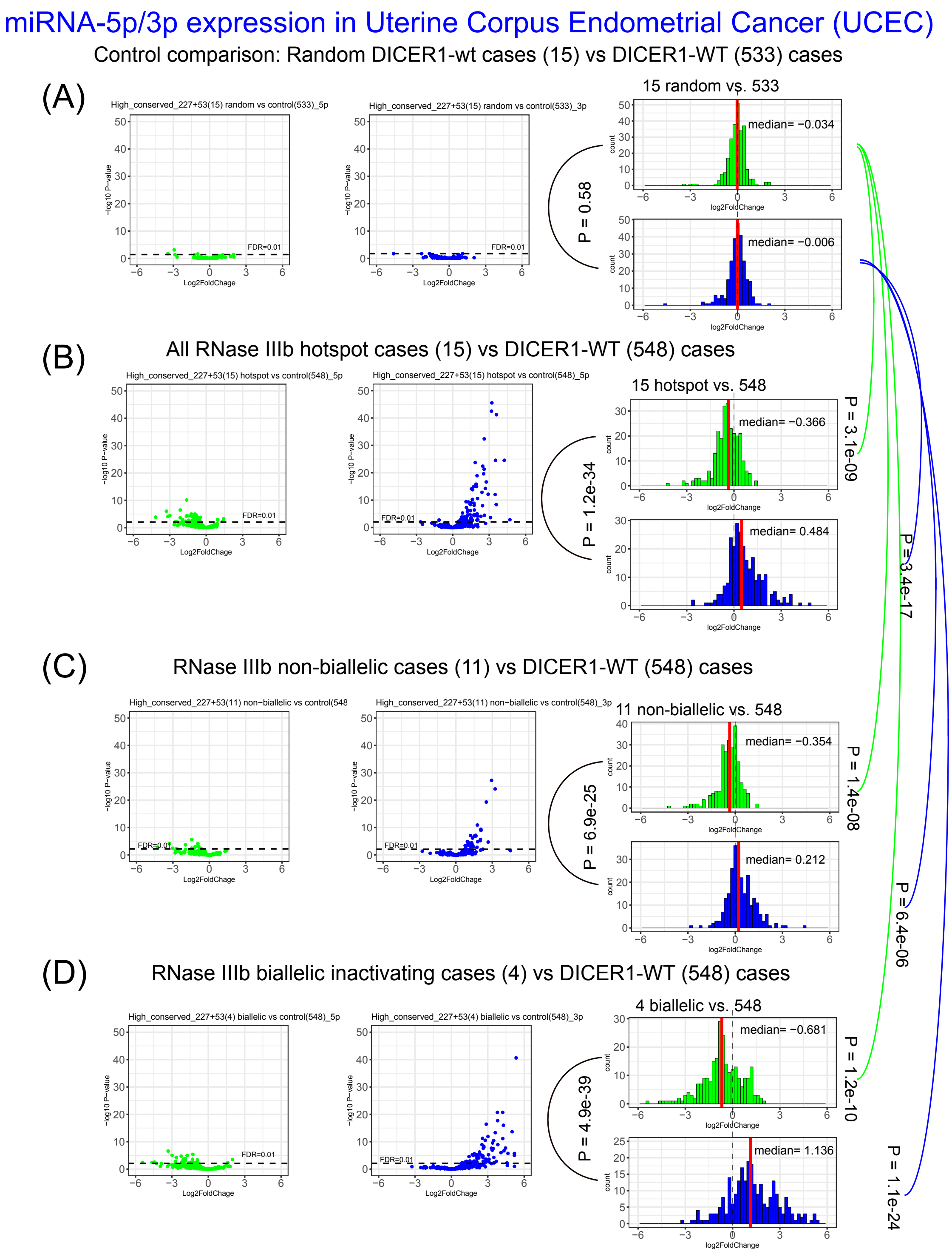

Supplementary Figure S4: miRNA-5p/3p expression in Uterine Corpus Endometrial Cancer (UCEC), showing miRNA processing asymmetry alterations across small RNA datasets. For all comparisons, a volcano plot, and a barplot are shown for miRNA-5p (green), and -3p (blue), respectively. (A) miRNA-5p/3p expression in randomly selected non-hotspot endometrial cases compared to remainder of DICER1-wt, in addition to other endometerial WT cases (hereafter, together referred as WT). In control comparisons some miRNAs exhibited apparent up/downregulation, but almost none were significant. On the other hand, when miRNA-5p/3p expression was compared between WT cases and either all RNAseIIIB mutations (15 cases) (B), or just the non-allelic RNase IIIB hotspot mutations (11 cases) (C), or only the biallelic hotspot mutations (4 cases) (D), we find significantly elevated miRNA-3p reads, and substantial reduction of miRNA-5p reads in all three comparisons. Of note, only biallelic hotspot mutations represented most drastic bias in miRNA-5p/3p experssion. False disovery rate for significantly different expression was determined using the Benjamini-Hochberg method at a FDR of 0.01. Other P-values for all comparisons are from a Wilcoxon Rank-Sum test.

Vedanayagam et al
Supplementary Figure 4

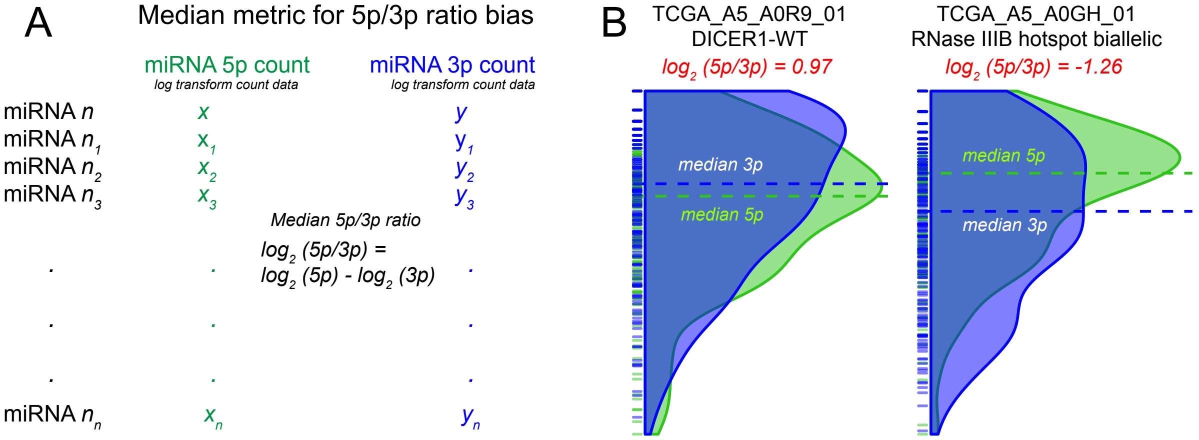

Supplementary Figure 5. Schematic and utilization of a scoring metric to evaluate relative production of miRNA-5p and -3p strands across a library.
(A) We focused on a set of 280 well-conserved and/or broadly-expressed miRNAs (see Supplementary Table 5). We summarize the log2 expression counts of all 5p and 3p strands separately. Because many miRNAs exhibit highly asymmetric maturation of 5p and 3p strands, we found it advantageous to calculate the median expression of 5p and 3p strands in a library, as opposed to the mean expression, which was more biased by highly expressed loci. The median 5p/3p ratio m^i_{53} is given as $\log_2(5p/3p)$.
(B) The shift in 5p:3p arm distribution between an arbitrarily selected DICER-wt sample and an RNase IIIb hotspot with a biallelic inactivating mutation is illustrated, with the resulting effect on the m^i_{53} metric.

Vedanayagam et al
Supplementary Figure 5

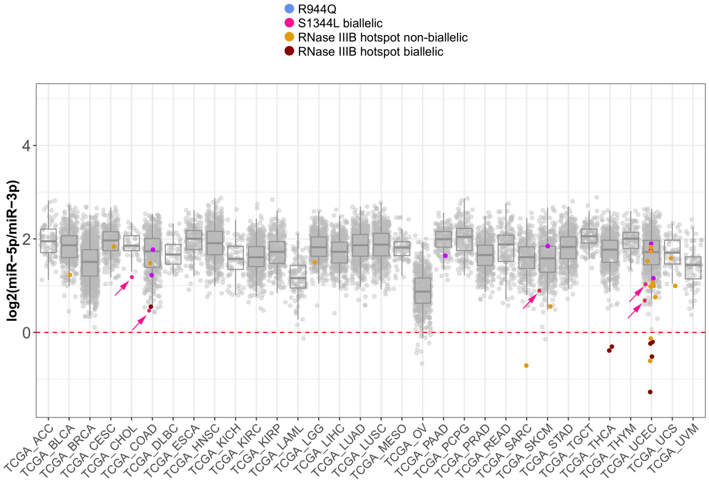

TCGA cancer type

LAML Acute Myeloid Leukemia
ACC Adrenocortical carcinoma
BLCA Bladder Urothelial Carcinoma
LGG Brain Lower Grade Glioma
BRCA Breast invasive carcinoma
CESC Cervical squamous cell carcinoma and endocervical adenocarcinoma
CHOL Cholangiocarcinoma
COAD Colon adenocarcinoma
ESCA Esophageal carcinoma
HNSC Head and Neck squamous cell carcinoma
KICH Kidney Chromophobe
KIRC Kidney renal clear cell carcinoma
KIRP Kidney renal papillary cell carcinoma
LIHC Liver hepatocellular carcinoma
LUAD Lung adenocarcinoma
LUSC Lung squamous cell carcinoma
DLBC Lymphoid Neoplasm Diffuse Large B-cell Lymphoma
MESO Mesothelioma
OV Ovarian serous cystadenocarcinoma
PAAD Pancreatic adenocarcinoma
PCPG Pheochromocytoma and Paraganglioma
PRAD Prostate adenocarcinoma
READ Rectum adenocarcinoma
SARC Sarcoma
SKCM Skin Cutaneous Melanoma
STAD Stomach adenocarcinoma
TGCT Testicular Germ Cell Tumors
THYM Thymoma
THCA Thyroid carcinoma
UCS Uterine Carcinosarcoma
UCEC Uterine Corpus Endometrial Carcinoma
UVM Uveal Melanoma

GBM Glioblastoma (Microarray data)

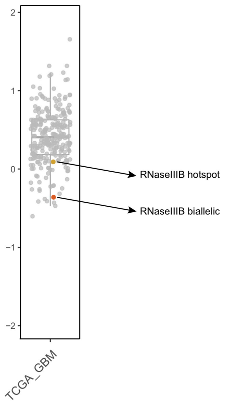

Supplementary Figure 6. Distribution of mi53 scores across TCGA samples segregated by cancer types. Bear in mind that the mi53 metric is influenced by miRNA expression. Thus, the overall averages for some cancers is lower than others; e.g., acute myeloid leukemia (LAML) and Ovarian serous cystadenocarcinoma (OV), Uveal Melanoma (UVM). Nevertheless, for the majority of cancers, the mi53 metric is relatively stable, allowing outliers to be defined. Note that many RNase IIIb hotspot mutants, well as the RNase IIIa hotspot (S1344L) mutants, are distinctly amongst the cases with the lowest mi53 scores in each cancer cohort. On the other hand, the seemingly recurrent, but functionally inert R944Q cases did not exhibit any strong bias. Moreover, the RNase IIIb hotspot mutants with biallelic inactivating mutations comprise the lower scoring cases amongst the entire ~10,000 TCGA cases analyzed. Glioblastoma (GBM) was the only legacy TCGA cohort only with microarray miRNA data available; thus there was limited power to analyze matching 5p/3p miRNA sets. However, an RNase IIIb case with biallelic inactivating mutation was amongst the lowest mi53 scoring cases in this cohort.

Vedanayagam et al
Supplementary Figure 6

A

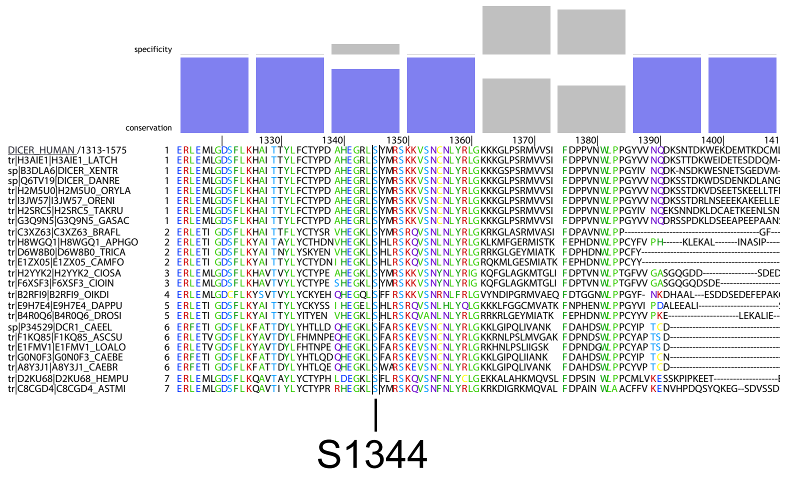

B

```
                        active site
                         1316  1320                       1344
hDICER RNase IIIa  NLERLEMLGDSFLKHAITTYLFCTYPDAHE---GRLSYMRSKKVSNCNLY
                    .:||||.||||:.|.:.||.:|   |.|.:   |.|:.:||..|:|....
hDICER RNase IIIb  CYQRLEFLGDAILDYLITKHL---YEDPRQHSPGVLTDLRSALVNNTIFA
                         1705  1709                       1733
                        active site
```

C DICER1 hotspot residues mapped onto RNase IIIb homodimer

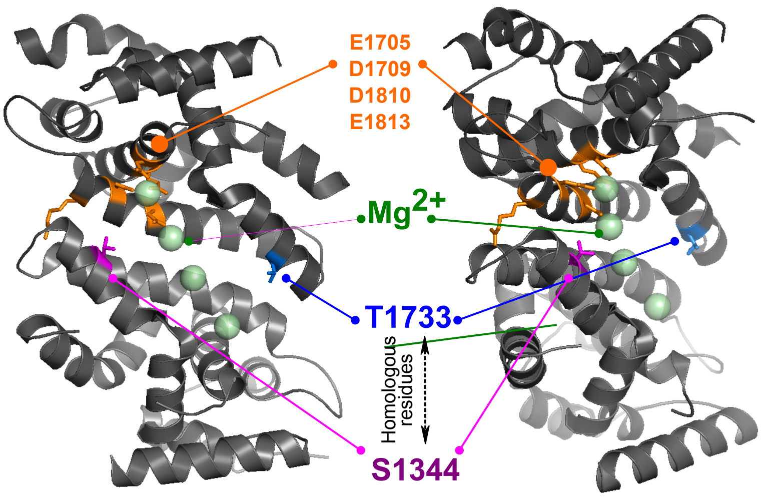

Supplementary Figure 7. Structural rationale for how RNase IIIa-S1344 affects RNase IIIb function.

(A) DICER RNase IIIa S1344 is highly conserved.
(B) DICER RNase IIIa S1344 corresponds to RNase IIIb T1733.
(C) Modeling based on a DICER1 RNase III homodimeric structure (PDB:2eb1) shows that cancer-prone S1344 in the RNase IIIa domain is homologous to T1733 in RNase IIIb domain, which in turn lies in close proximity to RNase IIIb metal ion-binding residues that form part of the active site (and are also cancer hotspot mutations. This provides evidence that S1344, although it resides within RNase IIIa domain, is important for proper function of the RNase IIIb domain.

Vedanayagam et al
Supplemental Figure 7
