# Supplementary material for: Cancer-associated mutations in DICER1 RNase IIIa and IIIb domains exert similar effects on miRNA biogenesis
Source: Nat Commun. 2019 Aug 15;10:3682. doi: 10.1038/s41467-019-11610-1 (PMC6695490; doi:10.1038/s41467-019-11610-1)
Supplement: Supplementary file 3 — Description of Additional Supplementary Files [file 41467_2019_11610_MOESM3_ESM.pdf]

Description of Additional Supplementary Files

File Name: Supplementary Data 1
Description: Summary of *DICER1* mutations in the TCGA PanCan dataset.

File Name: Supplementary Data 2
Description: Summary of *DICER1* mutations in the MSK-IMPACT dataset.

File Name: Supplementary Data 3
Description: Summary of m^{i}_{53} ratio across TCGA small RNA datasets.

File Name: Supplementary Data 4
Description: Evolutionary couplings analysis using human DICER1 aa 1271-1829.

File Name: Supplementary Data 5
Description: List of broadly expressed + conserved miRNAs used for comparing 5p and 3p populations in TCGA PanCancer dataset.
