# Supplementary material for: Cancer-associated mutations in DICER1 RNase IIIa and IIIb domains exert similar effects on miRNA biogenesis
Source: Nat Commun. 2019 Aug 15;10:3682. doi: 10.1038/s41467-019-11610-1 (PMC6695490; doi:10.1038/s41467-019-11610-1)
Supplement: Supplementary file 9 — Reporting Summary [file 41467_2019_11610_MOESM9_ESM.pdf]

naturereserch

Corresponding author(s): Eric C. Lai
Last updated by author(s): Jun 25, 2019

Reporting Summary

Nature Research wishes to improve the reproducibility of the work that we publish. This form provides structure for consistency and transparency in reporting. For further information on Nature Research policies, see Authors & Referees and the Editorial Policy Checklist.

Statistics

For all statistical analyses, confirm that the following items are present in the figure legend, table legend, main text, or Methods section.

n/a	Confirmed	
☐	☒	The exact sample size (*n*) for each experimental group/condition, given as a discrete number and unit of measurement
☐	☒	A statement on whether measurements were taken from distinct samples or whether the same sample was measured repeatedly
☐	☒	The statistical test(s) used AND whether they are one- or two-sided<br>*Only common tests should be described solely by name; describe more complex techniques in the Methods section.*
☒	☐	A description of all covariates tested
☐	☒	A description of any assumptions or corrections, such as tests of normality and adjustment for multiple comparisons
☐	☒	A full description of the statistical parameters including central tendency (e.g. means) or other basic estimates (e.g. regression coefficient) AND variation (e.g. standard deviation) or associated estimates of uncertainty (e.g. confidence intervals)
☐	☒	For null hypothesis testing, the test statistic (e.g. *F*, *t*, *r*) with confidence intervals, effect sizes, degrees of freedom and *P* value noted<br>*Give P values as exact values whenever suitable.*
☒	☐	For Bayesian analysis, information on the choice of priors and Markov chain Monte Carlo settings
☒	☐	For hierarchical and complex designs, identification of the appropriate level for tests and full reporting of outcomes
☒	☐	Estimates of effect sizes (e.g. Cohen's *d*, Pearson's *r*), indicating how they were calculated

Our web collection on statistics for biologists contains articles on many of the points above.

Software and code

Policy information about availability of computer code

Data collection	Data collection/Software used:<br>gdc-client tool (https://github.com/NCI-GDC/gdc-client) Data download tool<br>TCGA portal (https://portal.gdc.cancer.gov/) Database for TCGA PanCan data<br>cBioPortal (http://www.cbioportal.org/) Database for TCGA and MSK-IMPACT data<br>RSeQC (http://rseqc.sourceforge.net/) Data QC tool<br>miRBase (www.mirbase.org) miRNA annotation repository<br>featureCounts (http://subread.sourceforge.net/) miRNA counts tool<br>DEseq (https://bioconductor.org/packages/release/bioc/html/DESeq2.html) miRNA expression analysis<br>GDAC Firehose (https://gdac.broadinstitute.org/) TCGA level 1 database<br>AgiMicroRna (https://bioconductor.org/packages/release/bioc/html/AgiMicroRna.html) Processing and analysis of agilent microarray data<br>Evolutionary couplings (https://github.com/debbiemarkslab/EVcouplings) Evolutionary couplings analysis<br>TargetScan (http://www.targetscan.org/) miRNA targets database<br>MSigDB (http://software.broadinstitute.org/gsea/msigdb) Database for gene annotation sets for gene sets enrichment analysis<br>limma (https://bioconductor.org/packages/release/bioc/html/limma.html) RNA-seq analysis
Data analysis	The code for analyses conducted in this study and supplemental results for each of the analyses are available at https://github.com/dicerhotspot/.

For manuscripts utilizing custom algorithms or software that are central to the research but not yet described in published literature, software must be made available to editors/reviewers. We strongly encourage code deposition in a community repository (e.g. GitHub). See the Nature Research guidelines for submitting code & software for further information.

Data

Policy information about availability of data

All manuscripts must include a data availability statement. This statement should provide the following information, where applicable:

- Accession codes, unique identifiers, or web links for publicly available datasets
- A list of figures that have associated raw data
- A description of any restrictions on data availability

All relevant data are available from the authors. The source data underlying Figure 4 is now provided as a Source Data file. Somatic mutational data from the cohort described here is available in the cBioPortal for Cancer Genomics (http://cbioportal.org/).

Field-specific reporting

Please select the one below that is the best fit for your research. If you are not sure, read the appropriate sections before making your selection.

☒ Life sciences ☐ Behavioural & social sciences ☐ Ecological, evolutionary & environmental sciences

For a reference copy of the document with all sections, see nature.com/documents/nr-reporting-summary-flat.pdf

Life sciences study design

All studies must disclose on these points even when the disclosure is negative.

Sample size	All available genomic TCGA and MSK-IMPACT datasets were used where possible, and the sample sizes are noted throughout the study.
Data exclusions	All available datasets were used where possible. For specific tests, as described in the methods and paper, it was appropriate to exclude certain cancer cohorts. For example as the mi53 metric for miRNA arm strand is influenced by tissue-specific miRNAs, and we observed a global shift in this score in particular cancers, particularly ovarian serous cystadenocarcinoma cancer (OV), this cohort was excluded from certain analyses to separate this single large tissue-specific effect from the Dicer-mutant specific effect studied. As another example, since certain cancers are mutated in DNA repair pathways that result in large numbers of background mutations, it was appropriate to conduct some analyses with and without hypermutated tumor types (MSI/POLE). Analyses where exclusions were done have been well-described and experimentally rationalized.
Replication	The overall findings of this study were replicated between two large cancer genome sequencing cohorts, the TCGA and MSK-IMPACT data. For experimental tests (Northern blotting and luciferase sensor tests), at least two biological replicate experiments were done with qualitatively similar results as shown in the Figures.
Randomization	Samples were allocated according to genotypes. We performed control analyses using bulk matched cancer samples samples as well as random selected control samples that were matched in size to the mutation samples of interest, as well as by bootstrap analysis of random sampling from each tumor type.
Blinding	Blinding was not relevant to this study.

Reporting for specific materials, systems and methods

We require information from authors about some types of materials, experimental systems and methods used in many studies. Here, indicate whether each material, system or method listed is relevant to your study. If you are not sure if a list item applies to your research, read the appropriate section before selecting a response.

Materials & experimental systems

n/a	Involved in the study	
☐	☒	Antibodies
☐	☒	Eukaryotic cell lines
☒	☐	Palaeontology
☐	☒	Animals and other organisms
☒	☐	Human research participants
☒	☐	Clinical data

Methods

n/a	Involved in the study	
☒	☐	ChIP-seq
☒	☐	Flow cytometry
☒	☐	MRI-based neuroimaging

Antibodies

Antibodies used	anti-human Dicer (13D6, Abcam), 1:1000<br>β-tubulin (E7, DSHB) 1:1000

Validation	human Dicer antibody has been validated by loss of the Dicer antigen in human Dicer-KO hESCs (Teijeiro Stem Cell Reports 2018). ß-tubulin monoclonal has been extensively tested and validated (http://dshb.biology.uiowa.edu/tubulin-beta-_2) and produced reactivity of the expected molecular weight in our studies.

Eukaryotic cell lines

Policy information about cell lines

Cell line source(s)	Dicer-KO MEF cells were previously described (Yang et al PNAS 2010).
Authentication	Cells were authenticated by lack of mature miRNA production, and rescue by transfection of wildtype Dicer constructs, as demonstrated in other studies and in this study.
Mycoplasma contamination	Cells have been confirmed negative for Mycoplasma contamination as tested in 2018.
Commonly misidentified lines (See ICLAC register)	*Name any commonly misidentified cell lines used in the study and provide a rationale for their use.*

Animals and other organisms

Policy information about studies involving animals; ARRIVE guidelines recommended for reporting animal research

Laboratory animals	The study did not involve laboratory animals.
Wild animals	*Provide details on animals observed in or captured in the field; report species, sex and age where possible. Describe how animals were caught and transported and what happened to captive animals after the study (if killed, explain why and describe method; if released, say where and when) OR state that the study did not involve wild animals.*
Field-collected samples	*For laboratory work with field-collected samples, describe all relevant parameters such as housing, maintenance, temperature, photoperiod and end-of-experiment protocol OR state that the study did not involve samples collected from the field.*
Ethics oversight	*Identify the organization(s) that approved or provided guidance on the study protocol, OR state that no ethical approval or guidance was required and explain why not.*

Note that full information on the approval of the study protocol must also be provided in the manuscript.
